# 以维奈克拉为基础的方案治疗伴t（8;21）的急性髓系白血病12例疗效及安全性回顾性分析

**DOI:** 10.3760/cma.j.issn.0253-2727.2023.06.011

**Published:** 2023-06

**Authors:** 瑞华 米, 琳 陈, 琳 王, 昊 艾, 青松 尹, 旭东 魏

**Affiliations:** 郑州大学附属肿瘤医院/河南省肿瘤医院血液科，郑州 450008 Department of Hematology, the Affiliated Cancer Hospital of Zhengzhou University/Henan Cancer Hospital, Zhengzhou 450008, China

伴有t（8;21）染色体异常或RUNX1-RUNX1T1融合基因的急性髓系白血病（AML）预后相对较好，但老年或因严重合并症不适合强诱导缓解化疗的患者或患者出现难治/复发时如何选择诱导缓解治疗方案是临床的难题之一。维奈克拉（Ven）为一种口服的选择性小分子BCL-2抑制剂，其联合去甲基化药物（HMA）或低剂量阿糖胞苷（LD-Ara-C）成为75岁及以上、不适合强化疗的初治AML患者的治疗新选择[1]–[3]。目前以Ven为基础的方案治疗伴t（8;21）的AML患者疗效报道较少。本研究对我中心应用含Ven的方案治疗的伴t（8;21）的12例AML患者进行回顾性分析，探讨其临床疗效和安全性。

## 病例与方法

1. 一般资料：回顾性分析2019年6月至2022年9月郑州大学附属肿瘤医院收治的12例伴t（8;21）AML患者的临床资料，诊断根据2016版WHO造血和淋巴组织肿瘤的分型诊断标准[4]。所有患者经骨髓细胞形态学、白血病免疫分型、细胞遗传学及分子生物学等检查明确诊断。12例患者中，男7例，女5例，中位年龄52.5（14～70）岁，初治7例，1周期化疗后未缓解（NR）2例，多周期化疗后复发2例，难治性1例。治疗前骨髓原始细胞比例的中位数为44.75％（11.2％～75.0％），合并c-kit突变患者5例，其中D816V突变2例，非D816V突变3例，按照2022年欧洲白血病网络（ELN）AML危险度分层体系，12例患者均为预后良好组。患者的临床资料详见表1。

**表1:** 12例伴t(8;21)急性髓系白血病患者接受含维奈克拉方案治疗时的临床特征

例号	性别	年龄（岁）	FAB分型	骨髓象	染色体	基因突变	ELN预后分层
1	男	70	M_2_	增生明显活跃，异常中幼粒细胞74.4%	45,X,-Y,t(8;21)[10]	c-KIT(N820G+)，JAK2(exon14),ABCB1	低危
2	男	52	M_2_	增生活跃，异常中幼粒细胞66.0%	46,X,-Y,+4,t(8;21)(q22;q22)[10]	c-KIT(D816V+)	低危
3	男	57	M_4_	增生活跃，原始粒细胞31.2%，原始+幼稚单核细胞28.0%	45,X,-Y,t(8;21)(q22;q22)[10]	ASXL1/CEBPA（均不是Ⅰ类突变位点）	低危
4	女	35	M_2_	增生明显活跃，原始粒细胞27.0%	46,XX,t(8;21)(q22;q22)[9]/46,XX[1]	c-KIT（N822K+）,KDM6A(+)	低危
5	女	68	M_2_	增生明显活跃，异常中幼粒细胞54.5%	46,XX,t(8;21)(q22;q22)[10]	D816V(+)	低危
6	男	69	M_2_	增生活跃，原始粒细胞35.0%	46,XY[1]/46,XY,t(8;21)[7]/45,X,t(8;21),-Y[9]	D816Y(+)	低危
7	男	53	M_2_	增生明显活跃，异常中幼粒细胞75.0%	46,XY,t(8,21)(q22,q22)[20]	8exon，D419delGA	低危
8	男	18	M_2_	增生极度活跃，异常中幼粒细胞69.2%	46,XY,t（8,21）（q22)，i(17)(q10)[10]	CSF3R（+）	低危
9	女	14	M_2_	增生明显活跃，异常中幼粒细胞11.2%	46,XX,t(8,21)(q22,q22)[20]	ASXL1(+),EZH2(+),KRAS(+),EP300(+)	低危
10	男	45	M_2_	增生明显活跃，原始粒细胞17.2%	46，XY,t(8;21)(q22,q22)[8]/46,XY[2]	U2AF(+),ABCB1(+)	低危
11	女	56	M_2_	增生活跃，异常中幼粒细胞23.0%	46，XX，t(14;21) (q32;q22)t(8;21)(q22;q22){20}	ASXL1	低危
12	女	40	M_2_	增生活跃，异常中幼粒细胞18.0%	46，X,-X,t(8;21)(q22;q22)[5]/45,i dem,del(9)(q12q22)[3]/46,XY	阴性	低危

注 ELN：欧洲白血病网络

2. 治疗方法：Ven 100 mg第1天，200 mg第2天，400 mg 第3～28天，口服, 根据药物相互作用进行剂量调整。患者进入粒细胞缺乏（粒缺）期常规给予伏立康唑预防真菌感染，同时下调Ven剂量至100 mg/d；阿扎胞苷（AZA）75 mg/m^2^，第1～7天；高三尖杉酯碱（HHT）1 mg/m^2^ 第1～7天。骨髓抑制期HGB<60 g/L或出现临床症状时给予悬浮红细胞输注，PLT<20×10^9^/L时或有临床出血倾向时给予输注机制血小板，中性粒细胞绝对计数（ANC）<0.5×10^9^/L时常规给予G-CSF治疗，直至ANC>0.5×10^9^/L。

3. 疗效和不良反应：诱导治疗1个周期进行疗效评价，疗效评价标准参照文献[5]。其中初治unfit的标准：年龄≥75岁或18～74岁且至少符合以下条件之一：美国东部肿瘤协作组（ECOG）体能状态评分为2～3分；严重的心、肺、肝或肾脏疾病；存在任何医师判断不适合接受强化化疗的合并症[3],[6]。所有不良反应按照NCI常见不良事件术语标准4.0版进行报告和分级[7]。

4. 随访：采用电话、门诊随诊、医院病历登记系统等方式进行随访。随访截止时间为2022年9月30日。

5. 统计学处理：采用SPSS 24.0软件进行数据分析。结果数据以中位数（范围）或例数（％）表示。

## 结果

1. 疗效评估：初治7例患者中，接受VA方案治疗者5例，其中4例NR，1例完全缓解（CR）伴血细胞计数未完全恢复（CRi）；接受VAH方案治疗者2例，均获CR。接受VA方案治疗的5例患者不适合强化疗的原因：1例老年患者曾因主动脉夹层接受支架植入术，1例患者为“乳腺癌”治疗后继发急性白血病，另3例患者为合并活动性肺部感染。

2例复发患者，1例接受VA方案治疗，疗效评价NR，1例接受VAH方案治疗，疗效评价CR。

2例1个疗程IA方案诱导缓解治疗后NR的患者，均接受VAH方案，疗效评价均为CRi。

1例难治患者接受VAH方案治疗，疗效评价为CRi。具体疗效评估见表2。

**表2:** 12例伴t(8;21)急性髓系白血病患者的治疗经过及疗效评估

例号	既往治疗经过	疾病状态	VA或VAH方案	粒缺期（d）	1周期疗效评估	是否移植	是否生存
1	-	初治	VA	0	NR	否	是
2	-	初治	VAH	16	CR	否	否
3	-	初治	VA	14	NR	是	是
4	-	初治	VA	0	NR	是	是
5	-	初治	VA	26	NR	否	否
6	-	初治	VA	23	CRi	否	是
7	-	初治	VAH	15	CR	否	是
8	DA，HD-Ara-C×2，HA	复发	VA	0	NR	否	否
9	IA	化疗1周期NR	VAH	14	CRi	是	是
10	IA	化疗1周期NR	VAH	28	CRi	是	是
11	IA，ID-Ara-C，D-CAG	复发	VAH	13	CR	否	是
12	DA，D-CAG	难治	VAH	20	CRi	是	是

注 -：不适用；DA：柔红霉素+阿糖胞苷（Ara-C）；HD-Ara-C：大剂量阿糖胞苷；HA：高三尖杉酯碱+ Ara-C；IA：伊达比星+Ara-C；ID-Ara-C：中剂量阿糖胞苷；D-CAG：地西他滨+阿柔比星+Ara-C+ G-CSF；VA：维奈克拉+阿扎胞苷；VAH：维奈克拉+阿扎胞苷+高三尖杉酯碱；粒缺：粒细胞缺乏；NR：未缓解；CR：完全缓解；CRi：CR伴血细胞计数未完全恢复

2. 不良反应：截至2022年9月30日，12例患者中，例2、例5均后因疾病进展死亡，例8因1周期治疗NR后失访，其余9例患者均存活。

12例患者中，发生中性粒细胞减少9例（75.0％），粒缺期中位持续时间16（13～28）d，血小板减少10例（83.3％），贫血7例（58.3％）。最常见的非血液学不良反应为粒缺期发热（9例，75.0％），其他依次为肺部感染（4例，33.3％）、恶心（4例，33.3％）、呕吐（3例，25.0％），无重度感染的发生，所有患者均未见肿瘤溶解综合征的发生。

## 讨论

Bcl-2是一种抗凋亡蛋白，通过特异性靶向抑制Bcl-2蛋白和激活内源性线粒体凋亡途径引起肿瘤细胞的凋亡[1],[8]。Ven是一种口服的靶向Bcl-2的选择性抑制剂，临床前研究结果显示，其联合HMA或LD-Ara-C治疗AML患者疗效显著。

Ven联合HMA方案具有较高的CR率，Lou等[9]的研究纳入48例难治/复发性AML患者，联合用药的总有效率可达到47.9％，中位总生存（OS）时间为9.6个月。二者联合应用可能存在协同作用：AZA既可以降低MCL-1的表达，延缓Ven耐药性的出现，又能激活AML细胞中的BAX和线粒体凋亡，二者在体外协同杀伤白血病细胞，并在体内表现出联合抗肿瘤活性[10]。然而在临床实际应用中，Ven存在一定的耐药性，发生Ven耐药可能与下列因素有关：骨髓和外周血原始细胞比例低，单核细胞和中性粒细胞计数高，FAB分型中的M_4_、M_5_，存在KRAS、PTPN11、SF3B1基因突变[11]。关于Ven的耐药机制目前仍不明确，另有研究显示可能与BCL-2家族蛋白MCL-1以及BCL-XL的上调等多种因素有关[12]–[13]，但仍需进一步研究。

伴RUNX1-RUNX1T1融合基因的AML患者，预后相对较好，大多数患者会接受高强度化疗，然而对于具有合并症不适合高强度化疗的unfit患者或者难治/复发性AML患者，Ven的疗效如何？于文静等[14]报道，初治不适合强化疗的伴RUNX1-RUNX1T1（±c-kit D816突变）的AML患者6例，接受VA方案治疗后疗效评估达CR/CRi者仅2例；6例难治/复发性伴RUNX1-RUNX1T1（±c-kit D816突变）的AML患者接受VA方案治疗后疗效评估无一例达CR/CRi。本研究入组的12例患者中，初治unfit患者7例，VA方案的有效率（CR率+CRi率）为20％（1/5），2例采用VAH方案的患者全部有效；5例难治/复发或1个疗程NR AML患者中，1例采用VA方案者无效，4例采用VAH方案者全部有效，可见VA方案治疗伴RUNX1-RUNX1T1阳性的AML患者效果不佳，但在VA方案的基础上联合HHT，疗效明显提高。安全性方面，最常见的不良反应是骨髓抑制和感染，但均在可控的范围内，没有因不良反应导致死亡的发生。

Ven和HHT联合应用是否具有协同作用？Klanova等[15]报道，在弥漫大B细胞淋巴瘤（DLBCL）细胞株中，Ven和HHT联合应用，具有明显的协同杀伤活性，且在小鼠体内也得到证实。Yuan等[16]报道，HHT联合Ven，通过抑制MAPK/ERK和PI3K/AKT通路的激活，以及激活p53通路来协同促进AML细胞株和原代细胞的凋亡。

在伴RUNX1-RUNX1T1融合基因的AML患者中，VA方案疗效欠佳，但具体耐药的机制有待进一步研究；另外在VA方案的基础上联合HHT，疗效明显提高，可能与Ven和HHT联合应用，具有协同的抗白血病效应有关。鉴于本研究入组样本量少、随访时间短，所得结论有待进一步大规模、多中心、前瞻性的观察研究证实。

## References

[1] DiNardo CD, Pratz KW, Letai A (2018). Safety and preliminary efficacy of venetoclax with decitabine or azacitidine in elderly patients with previously untreated acute myeloid leukaemia: a non-randomised, open-label, phase 1b study[J]. Lancet Oncol.

[2] DiNardo CD, Pratz K, Pullarkat V (2019). Venetoclax combined with decitabine or azacitidine in treatment-naive, elderly patients with acute myeloid leukemia[J]. Blood.

[3] DiNardo CD, Jonas BA, Pullarkat V (2020). Azacitidine and venetoclax in previously untreated acute myeloid leukemia[J]. N Engl J Med.

[4] Arber DA, Orazi A, Hasserjian R (2016). The 2016 revision to the World Health Organization classification of myeloid neoplasms and acute leukemia[J]. Blood.

[5] 沈 悌, 赵 永强 (2018). 血液病诊断及疗效标准[M].

[6] Senapati J, Dhawan R, Aggarwal M (2021). Venetoclax and azacitidine (VenAZA) combination therapy in young unfit patients with AML: a perspective from a developing country[J]. Leuk Lymphoma.

[7] Wilson C NCI Common Terminology Criteria for Adverse Events (CTCAE) v3.0 and v4.0.

[8] Wei AH, Montesinos P, Ivanov V (2020). Venetoclax plus LDAC for newly diagnosed AML ineligible for intensive chemotherapy: a phase 3 randomized placebo-controlled trial[J]. Blood.

[9] Lou Y, Shao L, Mao L (2020). Efficacy and predictive factors of venetoclax combined with azacitidine as salvage therapy in advanced acute myeloid leukemia patients: A multicenter retrospective study[J]. Leuk Res.

[10] Jin S, Cojocari D, Purkal JJ (2020). 5-Azacitidine Induces NOXA to Prime AML Cells for Venetoclax-Mediated Apoptosis[J]. Clin Cancer Res.

[11] Zhang H, Nakauchi Y, Köhnke T (2020). Integrated analysis of patient samples identifies biomarkers for venetoclax efficacy and combination strategies in acute myeloid leukemia[J]. Nat Cancer.

[12] Niu X, Zhao J, Ma J (2016). Binding of Released Bim to Mcl-1 is a Mechanism of Intrinsic Resistance to ABT-199 which can be Overcome by Combination with Daunorubicin or Cytarabine in AML Cells[J]. Clin Cancer Res.

[13] Lin KH, Winter PS, Xie A (2016). Targeting MCL-1/BCL-XL Forestalls the Acquisition of Resistance to ABT-199 in Acute Myeloid Leukemia[J]. Sci Rep.

[14] 于 文静, 贾 晋松, 王 婧 (2022). 维奈克拉联合阿扎胞苷治疗急性髓系白血病的近期疗效: 单中心数据[J]. 中华血液学杂志.

[15] Klanova M, Andera L, Brazina J (2016). Targeting of BCL2 Family Proteins with ABT-199 and Homoharringtonine Reveals BCL2- and MCL1-Dependent Subgroups of Diffuse Large B-Cell Lymphoma[J]. Clin Cancer Res.

[16] Yuan F, Li D, Li G (2022). Synergistic efficacy of homoharringtonine and venetoclax on acute myeloid leukemia cells and the underlying mechanisms[J]. Ann Transl Med.

